# Robustifying the Deployment of tinyML Models for Autonomous Mini-Vehicles

**DOI:** 10.3390/s21041339

**Published:** 2021-02-13

**Authors:** Miguel de Prado, Manuele Rusci, Alessandro Capotondi, Romain Donze, Luca Benini, Nuria Pazos

**Affiliations:** 1 He-Arc Ingenierie, HES-SO, 2800 Delemont, Switzerland; romain.donze@he-arc.ch (R.D.); Nuria.Pazos@he-arc.ch (N.P.); 2 Integrated System Lab, ETH Zurich, 8092 Zurich, Switzerland; lbenini@iis.ee.ethz.ch; 3 DEI, University of Bologna, 40126 Bologna, Italy; 4 Department of Physics, Mathematics and Informatics, University of Modena and Reggio Emilia, 41121 Modena, Italy; alessandro.capotondi@unimore.it

**Keywords:** autonomous driving, tinyML, robustness, micro-controllers

## Abstract

Standard-sized autonomous vehicles have rapidly improved thanks to the breakthroughs of deep learning. However, scaling autonomous driving to mini-vehicles poses several challenges due to their limited on-board storage and computing capabilities. Moreover, autonomous systems lack robustness when deployed in dynamic environments where the underlying distribution is different from the distribution learned during training. To address these challenges, we propose a closed-loop learning flow for autonomous driving mini-vehicles that includes the target deployment environment in-the-loop. We leverage a family of compact and high-throughput tinyCNNs to control the mini-vehicle that learn by imitating a computer vision algorithm, i.e., the expert, in the target environment. Thus, the tinyCNNs, having only access to an on-board fast-rate linear camera, gain robustness to lighting conditions and improve over time. Moreover, we introduce an online predictor that can choose between different tinyCNN models at runtime—trading accuracy and latency—which minimises the inference’s energy consumption by up to 3.2×. Finally, we leverage GAP8, a parallel ultra-low-power RISC-V-based micro-controller unit (MCU), to meet the real-time inference requirements. When running the family of tinyCNNs, our solution running on GAP8 outperforms any other implementation on the STM32L4 and NXP k64f (traditional single-core MCUs), reducing the latency by over 13× and the energy consumption by 92%.

## 1. Introduction

Autonomous driving has made giant strides since the advent of deep learning (DL). However, scaling this technology to micro- and nano- vehicles poses severe functional and robustness challenges due to the limited computational and memory resources of the on-board processing unit [1,2]. Micro-controller units (MCUs) are typically used for small unmanned vehicles to balance the power budget of the sensing front-end and keep the system-energy low to extend the battery life.

Traditionally, small vehicles’ driving decisions have been off-loaded and carried out remotely, implying energy-expensive, long-latency, and unreliable transmissions of raw data to remote servers [3]. Off-system transfers can be prevented by processing data on-board and directly driving the motor controllers. However, given the battery-powered nature of the system, only a small fraction of the power can be allocated to the processing unit, i.e., the brain of the autonomous vehicle. Thus, compact and low-power machine learning (tinyML) techniques are needed to address these challenges and tackle on-device sensor data analysis at hardware, algorithmic, and software levels [4,5].

There exists a wide variety of machine learning (ML) techniques that span from traditional clustering, k-nearest neighbours, and decision tree methods [6,7,8], to modern deep neural networks, transformers, and GAN methods [9,10,11]. Convolutional neural networks (CNNs) represent the state-of-the-art for computer vision applications thanks to their efficiency in extracting patterns from input images and learning complex relationships.

However, a major challenge for the design of autonomous driving decision models is that the real-world environment changes over time: the data distribution used for offline training might not match the deployment environment’s underlying distribution—e.g., the car driving through different landscapes or lighting conditions. Hence, there is an increasing need to adapt to ever-changing environments to make vehicles more robust and efficient over time.

### 1.1. Goal Specification: Robust Low-Power Autonomous Driving

**Figure 1:**
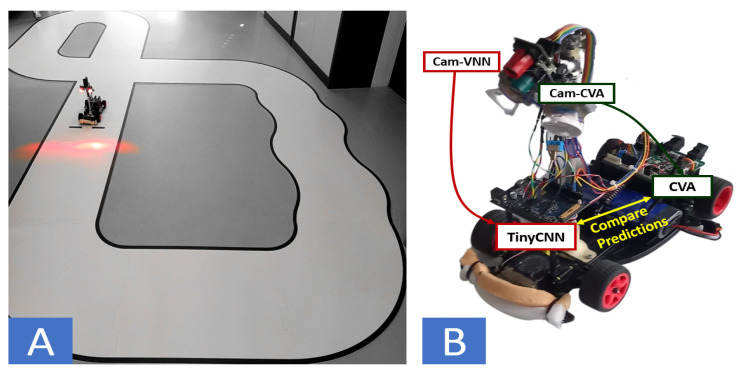
**Automotive application** use-case. (**A**) Mini-vehicle containing a linear camera and an on-board MCU running on circuit track. (**B**) Double-camera system setup for the close-loop learning task. One camera feeds the GAP8 (running a tinyCNN) and another feeds the NXP k64f (running the CVA).

We aim to shed light on the robustification of tinyML models for autonomous systems deployed in dynamic environments. We specifically focus on enabling the deployment of tinyCNNs to a low-power autonomous driving vehicle. We based our design on the NXP cup framework [12], an autonomous racing competition that offers a solid platform on which to test new ideas that can be reproduced or transported to other autonomous devices. Our vehicle consisted of a battery-powered mini-car that needed to autonomously issue seven different commands while driving on a small-scale racetrack: GoStraight, TurnLeft, TurnRight, CrossingStreets, StartSpeedLimit, and StopSpeedLimit, as shown in Figure 1A. The vehicle contained a linear camera—producing an image formed by a single line of pixels, giving a 1D grey-scale image—and an on-board NXP k64f MCU [13] to detect the current state, compute the required action, and drive the actuators accordingly.

The default autonomous driving controller is based on a conventional and handcrafted computer-vision algorithm (named CVA) that predicts accurately only under stable lighting conditions. The fragility to light condition is due to the nature of the CVA, as it calculates derivatives on the input image which requires good contrast (adding extra normalisation methods would cause a high latency overhead). The lack of robustness to lighting variability was countered by giving the camera a variable acquisition time, adapting to the environment’s conditions (controlled by a PID) to obtain clear images. However, the variability and slow-setting time of the camera adjustment (≥2 ms) limit the vehicle’s agility. Thus, we took a tinyML approach aiming to replace the conventional CVA with a tinyCNN to: (i) improve the robustness to lighting variations, and (ii) increase the performance, i.e., actions/sec, by learning challenging features from short and constant acquisition times.

### 1.2. Contributions

This paper presents an end-to-end flow of data, algorithms, and deployment tools that facilitate the deployment and enhance the robustness of a family of tinyCNNs to control an autonomous, low-powered mini-vehicle. We combined model compression and parallel computation to reduce the CNN’s inference latency, allowing us to keep up with the on-board camera at s faster sampling rate (1 ms) compared to the original CVA solution (2 ms). Despite the tinyCNN’s accuracy degradation due to low-contrast images, the prediction’s accuracy is recovered over time by applying an imitation learning (IL) methodology.

We implemented a system with two cameras, as shown in Figure 1B. The system couples a dedicated fast-rate camera (CAM-VNN) that feeds a multi-core processor (GAP8) running CNN inference, and a teacher system employing the CVA with access to a slower frame-rate camera (Cam-CVA), providing clearer images. We implement a closed-loop learning flow with the target deployment environment in-the-loop by comparing the outputs of both methods at runtime (tinyCNN vs. CVA). If the tinyCNN’s prediction differs from the CVA’s, the current sample of the fast-rate camera is sent to a remote central unit, creating an incremental dataset. We retrained the tinyCNN on the complete dataset, improving its prediction capabilities. Thus, when applying the closed-loop learning strategy, the most accurate tinyCNN model reached an accuracy of 97.4% on the fast-rate camera’s dataset, which is 16% more accurate than a bigger model (LeNet5) trained on the initial dataset.

Additionally, we propose an on-board ML predictor to select at runtime between a low-energy tinyCNN and a high-energy but more accurate tinyCNN based on the current image. This approach leads to energy saving by choosing the more energy-demanding CNN only for the more challenging inputs. Thus, our contributions are the following:

- We introduce a closed-loop learning methodology that enables the tinyCNNs to learn through demonstration. By imitating an expert with access to good-quality images, the tinyCNNs gain robustness to lighting conditions while having only access to a fast-rate camera, thereby doubling the system’s throughput compared to CVA.
- We leverage GAP8 [14], a parallel ultra-low-power RISC-V SoC, to meet the CNN inference computing requirements of this agile driving use case by adding it as a System-on-Module (SoM) on the NXP platform.
- We introduce an ML predictor that can swap the tinyCNN model at runtime to minimise energy consumption by analysing the input image. Thereby, we find new Pareto-optimal points where the combination of tinyCNNs consumes up to 3.2× less energy than always using the most accurate tinyCNN, while achieving only 3.2% less accuracy.

Further, we compare our deployment solution on GAP8 (50 MHz) with two platforms featuring an Arm Cortex-M processor: an STM32L4 (80 MHz) and an NXP k64f (120 MHz). We show the Pareto-optimal front where our solution dominates all other implementations, reducing the latency by over 13× and the energy consumption by 92%.

## 2. Related Work

We categorise the related work in three main areas:

### 2.1. Learning Methodology

There exists a wide variety of ML techniques that can be grouped into three classes: supervised learning, unsupervised learning, and reinforcement learning [15]. Supervised learning [16] corresponds to those methods that employ a labelled dataset or a teacher to guide the training phase. Examples of this class are k-nearest neighbours [7], support vector machine [17], decision trees [8], multi-layer perceptron, and convolutional neural networks [9]. Contrarily, unsupervised learning algorithms autonomously learn structures or patterns in the data without any teacher or labels. Examples of this class are clustering [6] and auto-encoders [18]. Finally, reinforcement learning (RL) [19] relies on the notion of an agent that makes decisions in an environment to maximise an objective or cumulative reward. Value-based algorithms such as Q-learning [20], and actor-critic algorithms such as A3C [21] are some examples of RL.

In this work, we focus on convolutional neural networks (CNNs), as they represent the state-of-the-art for computer vision. For the training of the CNNs, we introduce a learning flow that employs an imitation learning (IL) methodology. IL leverages the idea of a student learning from an expert that gives directives through demonstration [22,23]. Thus, the student has access to valuable data that can speed up the learning process and make it safer for methods deployed in real-life scenarios, e.g., autonomous driving. In this context, ALVINN [24] proposed a CNN-based system that, trained on driving demonstrations, learns to infer the steering angle from images taken from a camera on-board. Similarly, PilotNet [25] and J-Net [26] employed a system that collects the driver’s signal to label a training dataset with on-board cameras. However, these approaches only use the expert to label the training datasets.

Instead, we propose a closed-loop learning methodology where the learner confronts the expert in real drive and gradually improves through demonstration. In this direction, Pan et al. proposed [27], wherein they optimise the policy (online) of a reinforcement learning agent that imitates an expert driver with access to costly resources, while the agent has only access to economical sensors. However, their approach is not compatible with our use case where low-power systems cannot hold such levels of computation and memory.

In a different context, Taylor et al. [28] proposed an adaptive method (online) to change the CNN at runtime to improve the overall accuracy when tested in the ImageNet challenge and deployed on a Nvidia Jetson. However, their runtime predictor is implemented in python, which is rather slow (≈ 200 ms) and not suitable for MCUs. Inspired by this work, we implemented an optimised ML predictor that can swap the CNN at runtime to improve the energy efficiency of our autonomous low-power driving use case. Unlike studies such us [29], we do not intend to predict far-sighted forecasts, but we want to infer (predict) whether the current image will be well classified by a low-energy CNN, thereby saving energy. If the result of the prediction is “no,” a larger and more accurate CNN is used instead.

Several ML algorithms could be used to implement our predictor. For instance, a KNN algorithm was employed in [28] while an MLP and a SVM were used instead in [30,31] for micro-drilling and cancer genomics applications, respectively. Other works, such as [32,33], introduced self-learning or automated design space exploration methods to adapt to the current environment and find the best method. However, those methods require floating-point operations and large amounts of computation [19], restricting their use in MCUs. In this work, we evaluated several ML algorithms, trading off accuracy and latency. We put a strong focus on deployment optimisation and found that a DT gives the right balance, achieving a prediction in as little as 15 µs.

### 2.2. High-Performance Autonomous Driving

There exist multiple CNN approaches for autonomous driving [34], ranging from standard-size to small-scale vehicles. On the higher end, Nvidia and Tesla introduced PiloNet [25] and AutoPilot [35], requiring dedicated platforms such as TESLA FSD chip and NVIDIA drive, which provide TOPs of computing power and tens of gigabytes of memory for their large CNN solutions. Other approaches such as DeepRacer [36], F1/10 [37], and DonkeyCar [38] require GOPs and hundreds of megabytes that impose the use of high-end embedded platforms such as Nvidia Jetson, Raspberry PI, or Intel Atom. By contrast, we focus on end-to-end CNN solutions suitable for very low-power vehicles with MCUs featuring MOPs and up to a few megabytes, which is an unexplored field.

### 2.3. Low-Power DL deployment

On the deployment side, multiple software stacks have been introduced to gain flexibility for edge inference. Solutions targeting mid-high processors, such as ARM Cortex-A cores, run fast inference by making use of DSP units to accelerate the matrix multiplication kernels, which demands the most significant part of the DL workload [39,40,41]. However, these devices do not fit the energy requirements of battery-operated systems. In the context of resource-constrained MCUs, several software stacks have been introduced to address the severe limitations in terms of computational and memory resources. STMicroelectronics has released X-CUBE-AI to generate optimised code for low-end STM32 MCUs [42]. Similarly, ARM has provided the CMSIS-NN library [43], which targets Cortex-M processors and constitutes a complete backend for quantised DL networks exploiting 2x16-bit vector MAC’s instructions [44,45]. The functionality of the library has been demonstrated in several DL use cases running on MCUs [44,45]. Recently, the CMSIS-NN dataflow has been ported to a parallel low-power architecture, PULP, originating PULP-NN [46], which exploits 4x8-bit SIMD MAC instructions and achieves an up to 15.5 MAC/cycle on a parallel processor, such as the GAP8. In this work, we leveraged the GAP8 processor for the autonomous driving use case by adding it as an SoM on the NXP platform. The prediction produced by the GAP8, running CNN inference, is compared against the result produced by the NXP module, running the handcrafted computer-vision algorithm (CVA). In a case of mismatch, the camera input sample is transmitted to a remote server to enhance the dataset for offline training. MCU platforms’ memory limitations (typically a few MBs) prevent the entire dataset’s storage for online fine-tuning [47,48]. Moreover, an on-device learning procedure results in an expensive computational load, usually demanding floating-point engines that are not featured by ultra-low-power MCUs. Lately, some works have shown continual learning algorithms that do not require the storage of the full dataset for online fine-tuning [48,49,50]. However, these algorithms reach a lower accuracy with respect to retraining on the full enhanced dataset [51]. Thus, we have opted for an offline retraining methodology for our CNNs to gain robustness in this autonomous driving use case.

## 3. Initial Evaluation and Challenges

We aimed to take a tinyML approach and replace the initial CVA solution with a tinyCNN. First, we evaluated an initial setup and verify the challenge of porting DL methods on MCUs. In Section 4, we introduce the methods to enhance the performance and robustness of the deployed CNNs. Finally, in Section 5, we show the result of the several methodologies and compare three MCU platforms for the CNN’s inference latency and energy. Thus, we evaluated an initial setup to assess a CNN’s accuracy and performance in the target use case.

### 3.1. Data Collection

We have manually collected and labelled three initial datasets, each containing around 1000 samples per class (driving action) for the training set and 300 for the test set. The first dataset, Dset-2.0, contains samples with a fixed acquisition time of 2.0 ms—clear enough images—where the CVA can still predict well the required action. On the other hand, the second and third datasets, Dset-1.5 and Dset-1.0, hold more challenging samples (low contrast) with 1.5 and 1.0 ms acquisition times where the CVA fails to predict well, and thus, we aimed to use a CNN instead.

### 3.2. Training

We chose LeNet5 [52] for our initial evaluation, as it is a small and well-known CNN architecture, which is also used in [26]. We use PyTorch as a training environment with an cross-entropy loss function, a SGD optimiser, data augmentation, and dropout to avoid overfitting. Thus, we obtained an accuracy of 99.53% on the Dset-2.0 test set, but only 84.12% and 81.27% on the more challenging Dset-1.5 and Dset-1.0 test sets.

### 3.3. Lighting Condition Challenges

We aimed to show the effects of a dynamic environment on driving use cases, e.g., sudden sunlight or passing through a tunnel. We could emulate this scenario by training LeNet5 on the one of our Dsets, e.g., Dset-2.0, and testing it on Dset-1.0, as they contain the same elements but sampled with different acquisition times, and therefore, simulate different light conditions. As shown in Table 1, we can observe that the accuracy quickly drops when the light conditions differ from the learned distribution, as the features, e.g., line tracks, might be diffused or too shiny.

### 3.4. Deployment

We employed CMSIS-NN (INT8) as a backend to execute LeNet5 on the NXP k64f. The execution time turned out to be 5.4 ms, far too long compared to the ≈2 ms achieved by the conventional CVA on the same platform and conditions.

**Discussion:** Given the fragility of the CNN to lighting conditions and the long execution time of LeNet5, the initial CNN setup provides no benefit compared to the original CVA. To address these limitations, we propose a methodology to incrementally improve our DL-based approach under the latency-memory constraints of MCU devices, notably overcoming traditional CVA pipelines.

## 4. Robust and Efficient Deployment with tinyML

**Figure 2:**
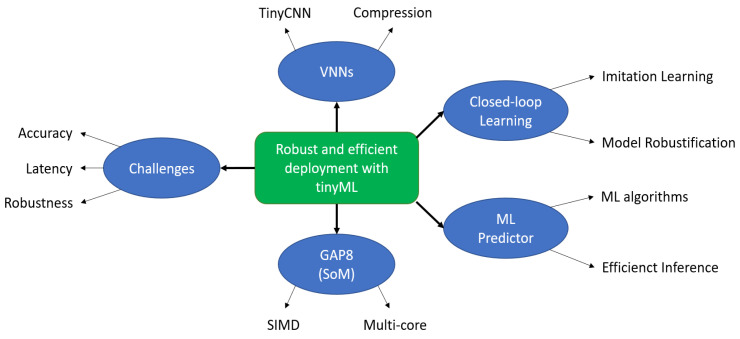
**Challenges and methodology** for robust and efficient deployment with tinyML for autonomous low-power driving vehicles.

To address the challenges discussed above, first, we created and compressed a family of tinyCNNs for efficient MCU deployment. Next, we introduce the GAP8 as a SoM to accelerate the CNN’s inference. Then, we detail the closed-loop learning methodology, and finally, we introduce the ML predictor to swap the tinyCNNs at runtime. The global methodology can be seen in Figure 2.

### 4.1. Vehicle Neural Network (VNN) Family

We modified LeNet5’s topology by varying the number of convolutional layers and the stride to shrink the model’s size, the number of operations, and the latency. Table 2 shows the different network configurations — networks and datasets have been open-sourced from https://github.com/praesc/Robust-navigation-with-TinyML (accessed on 30 December 2020). To have a higher tolerance to the diffusion of features in low-light conditions, we have opted for a relatively large kernel size (k = 5) for both the convolution and the pooling layers, giving the latter a stride of 3 to reduce the number of activations. As a result, we have created a family of tinyCNN called vehicle neural networks (VNNs) containing a range of layers that span from 1 to 3 convolutions followed by one fully-connected (FC) layer for the final classification. Overall, our family of VNNs achieved an accuracy between 91% and 98% on the Dset-2.0.

Training of CNNs is normally carried out using large floating-point data types. Such types need specific arithmetic units which may not even be present in MCUs due to their large area and energy consumption. Quantization reduces the storage cost of a variable by employing reduced-numerical precision. In addition, low-precision data types can improve the arithmetic intensity of the CNNs by leveraging the instruction-level parallelism, e.g., SIMD instruction. Thus, we have quantised our VNN models to fixed-point 8-bit to reduce memory footprint and power consumption [53,54]. We have employed post-training quantization where the weights can be directly quantised while the activations require a validation set (sampled from circuit runs) to determine their dynamic ranges. Thus, we observed a low accuracy loss (<3%) after the quantisation of the VNNs when trained on the initial Dsets and negligible loss (<1%) after the closed-loop learning phases.

### 4.2. System-on-Module Setup

We leveraged GAP8 [14], a parallel ultra-low-power RISC-V SoC based on the PULP architecture, to meet the CNN inference computing requirements. GAP8 features an MCU-system, which includes a RISC-V core, a large set of peripherals, and 512 kB of L2 memory, and an 8-core RISC-V (cluster) accelerator featuring a DSP-extended ISA that includes SIMD vector instructions, such as 4 × 8-bit Multiply and Accumulate (MAC) operations. Besides, the cluster is equipped with a zero-latency 64 kB L1 Tightly Coupled Data Memory and a multi-channel DMA engine for efficient data movements between in-cluster and off-cluster memories.

We added GAP8 as a System-on-Module (SoM) on the NXP platform and set up the system with two synchronised cameras (calibrated to have the same view of the circuit), one feeding the NXP and another feeding the GAP8 (see Figure 1B). We used the GAP8 as a CNN accelerator, which was only in charge of inferring the VNN on the input image, while the NXP controlled all the other sensors and actuators. The results from the VNN were constantly transferred via UART from the GAP8 to the NXP to drive the vehicle.

### 4.3. Closed-Loop Learning Flow

Initially, our family of VNNs achieved an accuracy of 78–83% on Dset-1.0, a drop of 15–20% compared to the VNNs trained on the Dset-2.0 setup. Thus, we needed a learning strategy to enhance the accuracy of the VNNs on the low-contrast images of the Dset-1.0 setup. However, developing robust applications on low-power systems require integrating data (often private), algorithms, and deployment tools, which might need significant expertise [55]. Hence, we propose a closed-loop learning methodology as a way to gradually improve the quality of our datasets and boost the accuracy of the VNNs.

**Figure 3:**
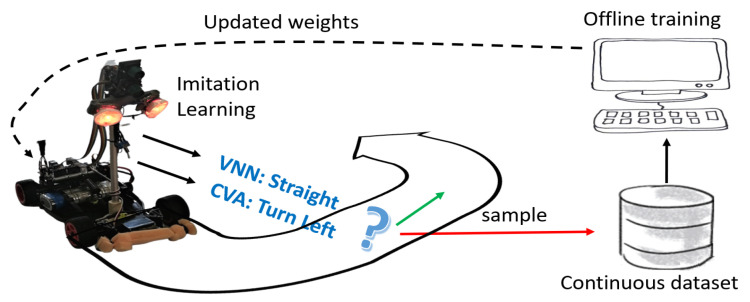
**Closed-loop learning pipeline.** End-to-end closed-loop learning cyclic methodology via imitation learning.

We implement this technique by collecting valuable data from the sensors at runtime, training the model from scratch on the cumulative set of data (offline), and pushing back the updates to the deployed VNN; see Figure 3. Thus, we pursue two main objectives: (i) improving the robustness to lighting variations and, (ii) increasing the performance, i.e., actions/sec, by learning challenging features from shorter acquisition times.

We leverage the CVA in our closed-loop learning methodology, as we have experimentally found that it predicts accurately provided adequate light conditions (not possible to benchmark CVA statically on our Dsets as it uses previous samples to predict the current one; i.e., it works on a continuous data stream). We can assume the predictions of the CVA as ground-truth and follow an imitation learning (IL) approach where the CVA acts as a teacher to help the VNNs learn better features. Hence, we decoupled the original system (single camera feeding the CVA) and propose a setup with two cameras:

- Cam-CVA: Set with a variable (and long) acquisition time that adapts to the lighting conditions (controlled by a PID) and always provides clear images. This camera feeds the CVA running on the NXP.
- Cam-VNN: Set with a short and constant acquisition time that captures the lighting variability of the environment. This camera feeds the VNN running on the GAP8.

By confronting the results of both algorithms while the mini-vehicle runs in a lighting-changing environment, we can improve the generalisation capacity of the deployed VNN. Moreover, we can also leverage IL to increase the VNNs’ accuracy on the more challenging Dset-1.5 and Dset-1.0 setups, which, in turn, improves the system’s throughput (actions/sec). We carry out the IL methodology as follows:

- Step 1. While number of samples <N:
  - Mini-vehicle drives on the circuit while predicting the required action. Each input image that leads the VNN’s predictions to differ from those of the CVA is collected.
  - Each sample gets automatically labelled by the CVA, which receives clear images from Cam-CVA, and it is sent over to the PC.
- Step 2. It includes the new samples in the training set to reinforce those classes where the VNN has failed.
- Step 3. The model is trained (offline) and the updated model is pushed back to the GAP8. Back to step 1.

The learning procedure can be repeated multiple times. However, we find that the frequency of new sample discovery decreases over time, thanks to the deployed solution progressively improving. Thus, we show an experiment with several phases, which we describe in Section 5.

### 4.4. Runtime Predictor for VNN Swapping (Online)

**Figure 4:**
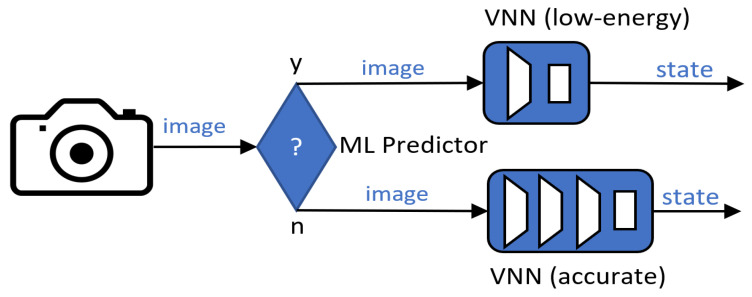
**Runtime predictor.** A VNN is chosen at runtime based on the input image.

We present an energy-efficient deployment use case where the VNNs trained on the closed-loop pipeline can be swapped at runtime trading accuracy and energy, as shown in [28]. We introduce a predictive method (predictor) in the system that continuously selects the VNN (architecture + weights) by analysing the input image before this is passed to the target VNN for inference; see Figure 4. Thus, we can leverage a low-energy VNN for “easy” images, and we employ a more accurate (and costly) VNN for more challenging samples.

Similarly to Taylor et al. [28], we opt to implement our predictor as a binary classifier. The binary predictor is trained to infer whether a given input image’s distribution will be well classified by a low-energy (and less accurate) VNN, e.g., VNN1 or VNN2. If the result of the prediction is negative, a larger and more accurate VNN, e.g., VNN3 or VNN4, is employed. We used same the training and test sets as in the closed-loop learning methodology to train and evaluate our binary predictor. We took one of our low-energy VNNs, and we performed inference on the dataset. We set apart those samples that were classified correctly from those that were not, creating our two categories, i.e., will-predict-well and will-not.

Since the predictor will cause an overhead in the system, we need to consider the predictor’s accuracy and performance. Thus, we trained and evaluate several machine learning (ML) algorithms to implement our predictor: a k-nearest neighbour (KNN), a support vector machine (SVM), a decision tree (DT), and a convolutional neural network (CNN).

## 5. Experimental Setup and Results

We present the results of our proposed methods introduced in Section 4 and represented in Figure 2 for the low-power autonomous driving use case. First, we show the effectiveness of the closed-loop learning flow to improve the robustness of a VNN. Next, we demonstrate the energy savings brought by the ML predictor. Then, we compare our solution running on GAP8 with two other traditional single-core MCUs, and finally, we summarise all the results achieved during this work:

### 5.1. Closed-Loop Learning Flow

We demonstrated the effectiveness of the proposed methodology on Dset-1.0, the most challenging setup, by applying the closed-loop learning strategy to improve the robustness of the model.

We show several stages, starting by training each VNN on the original Dset-1.0 training set. Next, we combined our three Dsets: 2.0, 1.5, and 1.0, and trained the VNNs on it to make it have access to a richer data distribution. Then, we deployed a VNN – we choose VNN1, as it features a small architecture and yet good potential for learning, but any other VNN can be deployed—on the mini-vehicle and set the acquisition time of Cam-VNN to 1 ms. We made the vehicle run in a varying-light environment while the VNN and CVA results were confronted. Those new samples where VNN failed were collected at every stage and were merged into a Dset-1.0 training set to make an incremental dataset that we show in three phases: +25%, +50%, and +100% (new samples with respect to the original Dset-1.0). In the last stage, i.e., +100%, we heavily altered the lighting conditions on the environment to improve the robustness of the VNN. We trained (following the training details of Section 3.2) the VNN from scratch at each phase for 1000 epochs on the complete set and sent an update of the weights to the vehicle before starting the next phase. We performed each training three times to account for the variability in the random initialisation.

**Figure 5:**
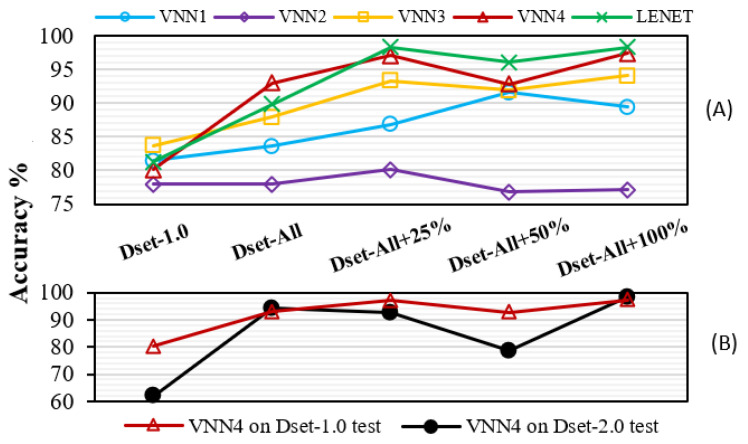
**Closed-loop learning evaluation.** X-axis is shared. (**A**) The VNN’s accuracy on Dset-1.0 test (8-bit). (**B**) VNN4 trained on Dset-1.0 learns/forgets features from Dset-2.0.

Figure 5A displays the results obtained on the Dset-1.0 test set. Initially, our family of VNNs achieved an accuracy of 78–83%. After training the VNNs on the combined dataset (Dset-All), most of the VNNs were able to generalise better, and their accuracy noticeably improved due to the richer diversity of light conditions. Further, when leveraging the closed-loop learning methodology through IL and training the networks on the reinforced dataset, VNN3, VNN4, and LeNet5 reached accuracies of 94.1%, 97.4%, and 98.3%. By contrast, VNN2’s accuracy remained mostly constant and VNN1’s decayed at the end, probably due to their shallow topology and lower capacity, failing to learn from more challenging data. Overall, the closed-loop learning approach allowed an increase in accuracy of over 15% on Dset-1.0, matching the accuracy of the conventional CVA on Dset-2.0 while doubling the throughput of the system.

Figure 5B illustrates how VNN4 learns and forgets features. We trained VNN4 on the data from Dset-1.0 setup, i.e., the same as in Figure 5A, and compare VNN4’s accuracy on Dset-1.0 and Dset-2.0 test sets, which contained samples with different acquisition times, and therefore lighting conditions. We can observe that, at first, when the VNN4 had only been trained on Dset-1.0 initial’s data, the accuracy on Dset-2.0 was poor. Nonetheless, when VNN4 received data from Dset-All, it generalised better and performed well in both test sets. However, as the experiment went on and VNN4 only saw data from the Dset-1.0 setup, it tended to forget and slowly decreased its accuracy on the Dset-2.0 test set. Finally, by heavily altering the lighting conditions at the 100% point, VNN4 can rehearse with data similar to those of Dset-2.0 and "remembers." This experiment depicts a challenge typical of continuous learning. It shows signs of the effects of catastrophic forgetting [47] and how rehearsal techniques can tackle this issue [48], which we plan to address in the future.

### 5.2. Runtime Predictor for VNN Swapping

In this section, we discuss the runtime predictor’s results to reduce energy consumption. First, we evaluate several ML algorithms used to implement the predictor and then we show the energy and accuracy results when it is integrated into the system.

#### 5.2.1. Evaluation of ML Algorithms

For the evaluation, we employed an open-source framework [56] that offers implementations of the ML algorithms (KNN, DT and, SVM) in python (scikit-learn library) and C-code, which we used for training and deployment, respectively. Since the evaluation framework offers the implementations using float32 (FP) data type, we executed them on the NXP k64f MCU, which contains an FP unit. For the CNNs, we employed PyTorch and CMISIS-NN instead.

The ML models may take the input image directly or have a feature reduction step to decrease the number of input features. In case of a reduction, we can either (i) split the image into the three main parts of the driving track, i.e., left line, middle, and right line, and calculate the mean intensity of each part (based on experience) or (ii) employ principal component analysis (PCA) where we select the number of final features (maximum likelihood estimation (M), 3, 2, or 1). As for the CNNs, we developed two networks (Conv-Relu-Pooling and FC) following the same concepts of the VNNs but smaller in size to provide a fast response.

**Figure 6:**
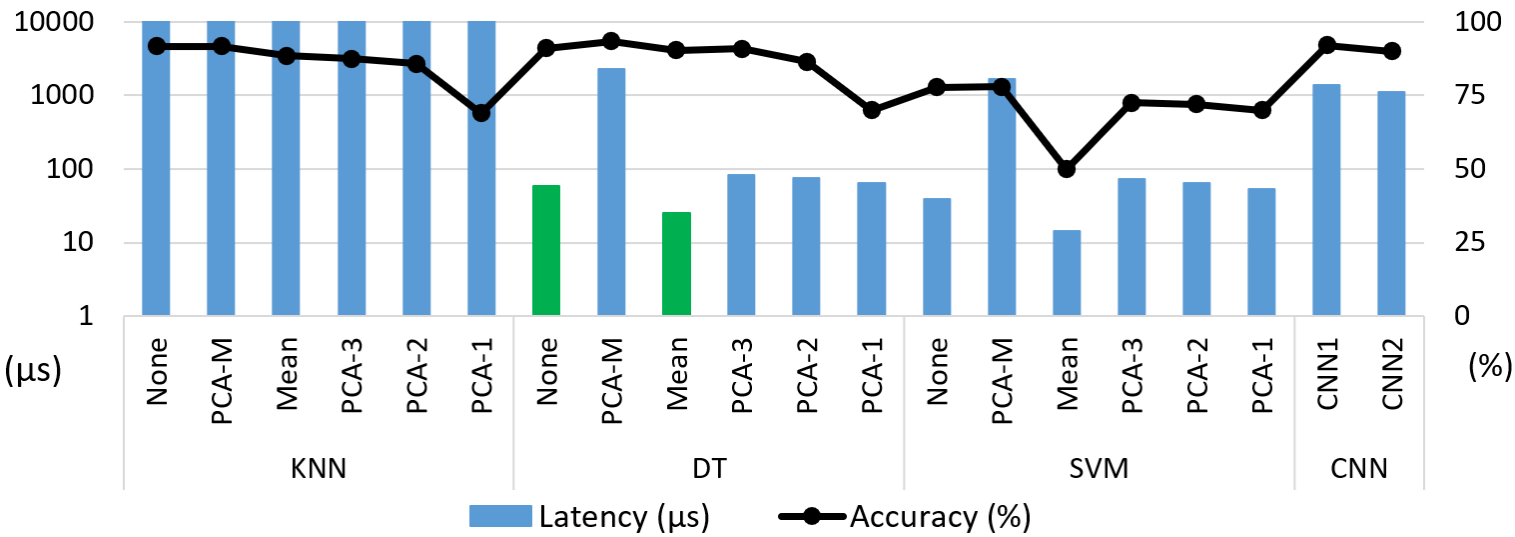
**ML predictors (latency vs. accuracy).** Binary classifier to infer whether VNN2 will classify the input images correctly. Algorithms contained float32 data types. Tested on NXP k64f MCU.

We evaluate the ML algorithms with different feature reduction possibilities, giving the VNN2–VNN4 swap as an example, since they are the lightest and most accurate networks, respectively. Thus, we trained our ML algorithms to infer whether VNN2 will predict the input image correctly in the Dset-1.0 setup. Figure 6 shows the accuracy and performance of the binary predictor. Overall, we observed that KNN and DT achieved high accuracy (>90%), while SVM’s accuracy fell under 80%. Both KNN and DT worked better with a large number of input features, i.e., no feature reduction (none) or "M," losing precision towards "PCA-1." The highest accuracy was achieved by DT-M with 93.5%. The ultra-tiny CNNs took the input directly (none) and achieved an accuracy of up to 92%, being on par with KNN and DT.

Regarding the performance, KNN is extremely slow as it needs to go through the whole training set to calculate the distance for each prediction. Additionally, CNN and PCA-M methods took a considerable amount of time on the inference and feature extraction steps, respectively. Thus, taking into account the performance and accuracy, we further evaluated DT-none and DT-mean (in green), which we converted to integers for better deployment on the GAP8 platform. Besides, we optimised the execution by embedding the pre-processing in the ML algorithm’s body, halving the memory accesses. In terms of clock cycles, DT-none and DT-mean became 10×, 2.5× faster than the FP counterpart, respectively. The speedup differs among them probably due to the feature extraction differences. Thus, we selected the DT-none algorithm as our predictor with an accuracy of 91.1% and a latency of 15 µs, making its overhead negligible with respect to the VNN’s inference time.

#### 5.2.2. VNN Swap Results

Next, we integrated our ML predictor into the system as shown in Figure 4 and evaluated it on Dset-1.0 test set. The ML predictor selected VNN2 85.8% of the time, leaving VNN4 for the rest, 14.2%. Thus, while VNN2 and VNN4 individually obtained accuracies of 80.2% and 97.4% on Dset-1-0 test set, the combined model accomplished 94.2%, 3.2% less accuracy than VNN4 but consuming 3.2× less energy, or 14% more accuracy than VNN2 with 1.5× more energy consumption (on the GAP8). By these means, we obtained a new point in the design space whereupon the combined model achieved an accuracy similar to VNN3’s while it consumed energy equivalent to that used by VNN1. We repeated the same experiment with a VNN1–VNN4 swap. In this case, the ML predictor achieved an accuracy of 87%, making the combined model accomplish 96.2% accuracy, 1.2% less than VNN4 but consuming 2.5× less energy.

**Figure 7:**
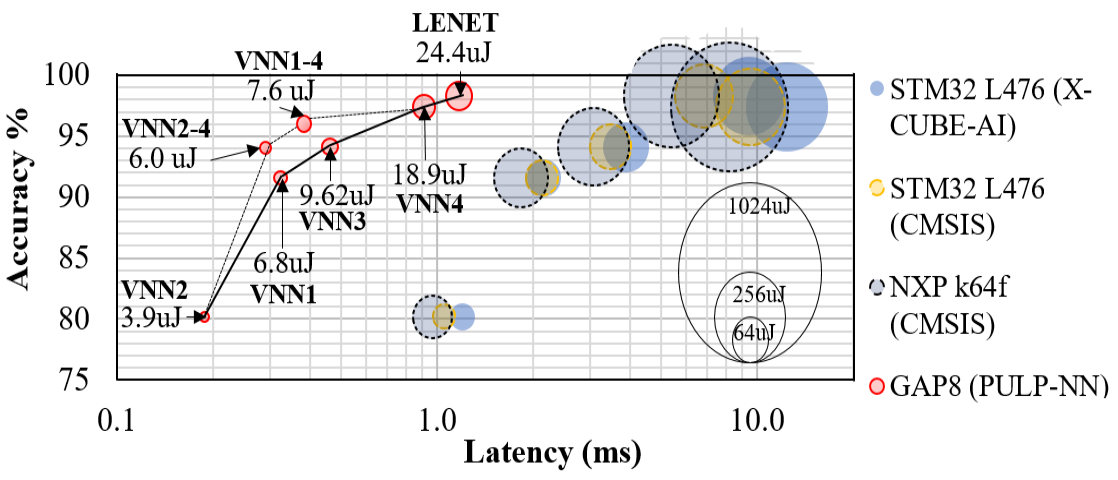
**Accuracy–latency–energy trade-off.** Accuracy (with respect to Dset-1.0, y-axis), latency (x-axis), and energy-consumption (balloon area) for an inference of the VNNs on the STM32 L476, NXP k64f, and GAP8. The black line highlights the Pareto front. The ML predictor achieves a new Pareto-optimal front for GAP8 by swapping VNNs at runtime.

Figure 7 summarises the accuracy, latency, and energy measured for the VNNs on several platforms. Looking at the GAP8 results, we can see that, overall, these combinations achieved reductions in energy and latency, pushing the Pareto-optimal front on the GAP8 towards higher efficiency (latency and energy calculated as weighted averages of both models).

### 5.3. MCU Efficient Deployment

In this section, we offer a comparison between different MCUs when running our VNNs. We compare the GAP8 (at 50 MHz) with two other classes of MCUs: a low-power single-core MCU (STM32 L476 at 80 MHz [57]), and a high-performance single-core MCU (NXP k64f at 120 MHz [13]). We also compare different inference backends supported on these devices, such as STMicroelectronics X-CUBE-AI [42], ARM CMSIS-NN [43], and PULP-NN [46] for GAP8.

#### 5.3.1. MCU Hardware/Software Inference Evaluation

**Figure 8:**
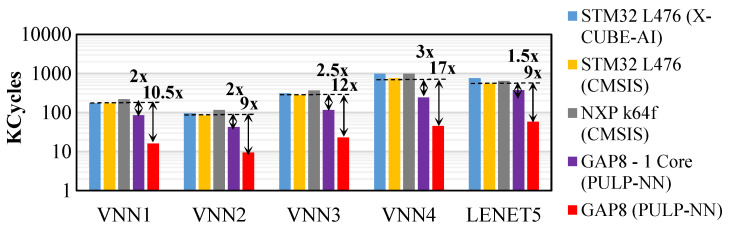
**VNN inference performance**. Kilo clock cycles spent on the different MCUs evaluated in this work for all the VNN family (8 bit).

Figure 8 reports the inference comparison, in terms of clock cycles. We compare our optimised solution, the GAP8 platform coupled with the PULP-NN backend, against an STM32 L476 MCU supporting either X-Cube-AI [42] or CMSIS-NN [43], and an NXP k64f MCU coupled with the CMSIS-NN [43]. First, we evaluate X-Cube-AI and CMISIS-NN on the same platform to have an estimation of the performance of each backend. Thus, when deploying the family of VNNs on the STM32 L476, we observed that X-Cube-AI backend was up to 27.8% slower than CMISIS-NN. Hence, we took the STM platform coupled with CMISIS-NN as a reference for comparison with the NXP and GAP8 platforms.

Thus, we observed that while the NXP solutions were up to 28% slower than STM’s when inferring the VNNs, the GAP8, with only one active core, reduced the clock cycles up to 3.13× thanks to its dedicated 4 × 8-bit SIMD MAC instructions. We obtained further speedups by using the 8-core cluster of GAP8, which lead to a further improvement of up to 6.4×. We argue that the discrepancy from the ideal 8× speedup is related to the low workload of small networks for which the parallelization overhead is not negligible. Overall, running an inference task on the GAP8’s cluster can be over 21× faster than the NXP or STM32 (X-Cube-AI) solutions.

#### 5.3.2. Energy–Accuracy–Latency Trade-off

Thanks to the high accuracy obtained through the closed-loop learning methodology, we can employ a camera with a short-acquisition time (1 ms), which we set as our latency target for the classification task. Figure 7 summarises the accuracy, latency, and energy measured on the different MCUs. All VNNs deployed on GAP8 met the 1 ms target and established the accuracy–latency Pareto frontier, dominating all the other implementations on STM32 L476 and NXP k64f. Remarkably, only VNN2 got under 1 ms on the NXP k64f (0.97 ms) due to its shallow topology and the higher clock frequency of the NXP.

Looking at the energy consumption of a single classification, VNN4 on GAP8 (8 cores, 50 MHz) consumed 18.9 μJ, 65.2% less compared to VNN2 on NXP k64f (120 MHz) while being 17.2% more accurate. The same VNN2 on GAP8 consumed 3.9 μJ, 92.8% less than the NXP k64f. The usage of STM32 L476 (80 MHz) is only possible if executing VNN2 and relaxing the target latency up to 1.5 ms. VNN2 on STM32 L476 consumed 66.26% less energy (18.3 μJ) compared to the NXP k64f using the same network topology and the same amount of energy as VNN4 on GAP8, while the latter caused 13.5% more latency and 17.2% more accuracy.

### 5.4. Robust and Efficient Deployment with tinyML Results

Table 3 summarises the results achieved through the different methodologies and optimisations introduced in the paper. Firstly, we can observe that by using the GAP8 platform for CNN deployment, we obtained faster and more energy-efficient solutions; e.g., LeNet5’s deployment became 4.5× faster and consumed 12.4× less energy than the NXP baseline. The improvement of GAP8’s solution over NXP’s is explained by the use of GAP8’s multi-core acceleration and the optimized NN SW stack that efficiently exploits SIMD 8-bit MAC instructions. Nonetheless, the inference model’s accuracy (LeNet5) is limited to 81.3% because of distribution shifts between the training and deployment environments. To overcome this issue, we enhanced the training dataset by gradually collecting more challenging samples that correspond to inference’s failures when the CNN is deployed in the target environment.

Thanks to our closed-loop learning methodology, we can train CNNs that learn from richer data distributions, improving the accuracy over time. That way, LeNet5 achieved a final accuracy of 98.3%, that is, an improvement of over 15%. Nonetheless, LeNet5 is still rather large and energy-hungry for our automotive agile use case. Thus, we propose a family of tiny CNNs (named VNNs) to replace LeNet5 without compromising accuracy. The tiny models are 1.3–6× faster (see Figure 7) and feature a 12–150× reduction in the number of parameters with respect to LeNet5. Lastly, we employed an ML predictor to swap the VNNs at runtime based on the input data. A lightweight inference model (VNN1) is usually used unless the input sample is marked as "challenging" by the online predictor. In that case, the sample is processed instead by the more complex and accurate VNN4 model. This latter strategy leads to extra savings in energy consumption (2.5×) with only a small reduction in accuracy (1.2%).

We believe that techniques such as self-learning or online learning would further improve the proposed methodology by (1) removing the need for a host system and all the required interconnections, and (2) adapting to the environment continuously. However, these techniques required large computational and memory requirements that extra-low-power MCUs cannot currently hold. We leave the exploration of these techniques as future work.

## 6. Conclusions and Future Work

We have shed light on the robustification of tinyCNN models deployed in a low-power driving use case (image classification task). We have introduced a closed-loop learning methodology that enables the tinyCNNs to imitate an expert, improving their robustness to lighting conditions in the target deployment scenario. Besides, the tinyCNNs can learn features from a fast-rate camera, doubling the system’s throughput with respect to the original computer-vision algorithm. To meet the CNN’s inference latency requirements, we have proposed a parallel ultra-low-power platform, which consumes as little as 3.9 μJ for a single inference. Finally, we have introduced a online predictor to swap the CNN at runtime, minimising the energy consumption by up to 3.2× with a modest drop in accuracy. Overall, we envision that this methodology can be applied for other autonomous use cases, e.g., drones, to improve the robustness and efficiency of tinyML methods. Nonetheless, the supervision signal for the imitation learning methodology should be adapted to each use case.

As future work, we aim to perform the CNNs’ training on-chip towards a continuous learning scenario, removing the need for a host system (and all the interconnection that it involves) to train the CNNs offline. Besides, it would also offer the possibility to adapt to the environment continuously.

## Figures and Tables

**Table 1:** **Lighting conditions challenges.** Networks were either trained on Dset-2.0 or Dset-1.0 and evaluated on all three Dsets.

Trained		Dset-2.0			Dset-1.0	
**Tested**	**Dset-2.0**	**Dset-1.5**	**Dset-1.0**	**Dset-1.0**	**Dset-1.5**	**Dset-2.0**
LeNet5 (%)	99.5	83.3	32.8	81.3	61.0	42.7

**Table 2:** Vehicle neural network (VNN) family.

	LeNet5	VNN4	VNN3	VNN2	VNN1
# Parameters (K)	72.85	6.04	0.97	1.29	0.48
Complexity(KMAC)	181.25	163.41	28.69	5.82	7.5

**Table 3:** Robust and efficient deployment with tinyML results on Dset-1.0.

	Baseline (LeNet5-NXP)	GAP Deployment (LeNet5-GAP)	Closed-Loop Learning (VNN4-GAP)	ML Predictor (VNN1/4-GAP)
Accuracy (%)	81.3	81.3	97.4	96.2
Latency (ms)	5.4	1.2	0.91	0.37
Energy (μJ)	302	24.3	18.9	7.6

## Data Availability

Data sharing not applicable.
